# Alcohol in urban streetscapes: a comparison of the use of Google Street View and on-street observation

**DOI:** 10.1186/s12889-016-3115-9

**Published:** 2016-05-26

**Authors:** Chris Clews, Roza Brajkovich-Payne, Emily Dwight, Ayob Ahmad Fauzul, Madeleine Burton, Olivia Carleton, Julie Cook, Charlotte Deroles, Ruby Faulkner, Mary Furniss, Anahera Herewini, Daymen Huband, Nerissa Jones, Cho Wool Kim, Alice Li, Jacky Lu, James Stanley, Nick Wilson, George Thomson

**Affiliations:** Department of Public Health, University of Otago, Wellington, PO Box 7343, Wellington New Zealand

**Keywords:** Alcohol, Marketing, Google Street View, Health promotion

## Abstract

**Background:**

Alcohol-related harm is a major global health issue, and controls on alcohol marketing are one intervention utilized by governments. This study investigated the use of Google Street View (GSV) as a novel research method for collecting alcohol-related data in the urban environment.

**Methods:**

The efficacy of GSV and on-street observation by observer teams was compared by surveying 400 m stretches of 12 streets in Wellington, the capital city of New Zealand. Data on alcohol sale, alcohol-related advertising, health promotion materials, regulatory information and visible alcohol consumption were collected.

**Results:**

A total of 403 retailers with evidence of alcohol sales and 1161 items of alcohol-related communication were identified in on-street observation. Of the latter, 1028 items (89 %) were for alcohol marketing and 133 (11 %) were for alcohol-related health promotion and alcohol regulation. GSV was found to be a less sensitive tool than on-street observation with only 50 % of the alcohol venues identified and 52 % of the venue-associated brand marketing identified. A high degree of inter-observer reliability was generally found between pairs of observers e.g., for the detection of alcohol retail venues the intra-class correlation coefficient (ICC) was 0.93 (95 % CI: 0.78 to 0.98) for on-street observation and 0.85 (95 % CI: 0.49 to 0.96) for using GSV.

**Conclusions:**

GSV does not seem suitable for the comprehensive study of the influences on alcohol consumption in the urban streetscape. However, it may still have value for large, static objects in the environment and be more time efficient than traditional on-street observation measures, especially when used to collect data across a wide geographical area. Furthermore, GSV might become a more useful research tool in settings with better image quality (such as more ‘footpath views’) and with more regularly updated GSV imagery.

## Background

There is evidence to indicate that exposure to various types of environmental contexts and imagery impacts on human health outcomes [1–3]. Of the many ways that urban environments affect these outcomes, visual stimuli can come from both the built and social environments, and can affect unhealthy behaviors [3–5].

This study focused on the visual aspects in urban streetscapes of alcohol-related items and behavior. Alcohol misuse has a significant population harm impact, and is the sixth leading risk factor for health loss globally (99 million disability-adjusted life-years lost in 2013) [6]. There is evidence from longitudinal cohort studies in different countries showing an association exists between the exposure of youth and young adults to alcohol advertising and media, and subsequent drinking behavior [7–9]. However, assessing people’s exposure to such environmental determinants of health remains a challenge in public health research [10, 11]. Traditionally, assessments of environmental determinants have been carried out in person through on-street observations, although new research tools are now being used to gather data on these variables [10–13].

Google Street View (GSV) has attracted attention as a potentially viable method for conducting street segment surveys online instead of using on-street observers [11]. GSV is a freely available technology that provides users with panoramic photographic images collected by data collection vehicles from many points along many streets worldwide [14]. It has been used as a research tool to study a wide range of public health topics, including smokefree signage, the built environment, cycling routes to school, and neighborhood characteristics [15–18]. These studies have found GSV to be resource efficient, unobtrusive, low cost, and a safe tool for researchers [11, 19]. Some of the limitations which have been noted by researchers include variations in the frequency of GSV image capture, the time of day of image capture and problems in detecting items in the environment [11, 18]. Despite its limitations, the existing literature has reported GSV to be effective in assessing some aspects of the built environment, and so its methodological viability to assess environmental determinants should be further explored.

Exposure to alcohol-related imagery may have significant public health implications, but there does not appear to be any published literature on GSV being used to gather visual information on such exposure. Furthermore, there have been few studies specifically measuring the extent of alcohol imagery and venues in the urban streetscape [20, 21].

Exposure to alcohol-related imagery is a suitable illustrative example to further explore the methodological utility of GSV in public health research. Firstly, in the existing literature, quantitative studies aiming to examine the extent of alcohol exposure in the streetscape have utilized standardized field observations as a method to measure alcohol cues [20], rather than utilizing innovative new technologies to measure these cues. Secondly, alcohol-related harm is a major public health issue worldwide, and the use of this novel research method may allow the assessment of environmental health determinants quickly and effectively across large geographical regions. Such virtual surveying may also reduce research costs by avoiding the necessity of travel to survey destinations. Therefore, this study aimed to explore the efficacy of GSV compared to on-street observation in assessing environmental health determinants, using alcohol as an illustrative example.

## Methods

### Street segment selection

Twelve street segments in Wellington, New Zealand’s capital city, were selected for collecting alcohol imagery data in the urban streetscape (see http://www.otago.ac.nz/wellington/otago114172.pdf for the 12 segments). They were within six km of the researchers’ workplace. Six suburban streets, in areas with a range of levels of area deprivation, and six central business district (CBD) streets were purposefully selected to cover a broad range of suburban areas of different socioeconomic status (SES), and a range of areas within the central business district. Street segments within the CBD were chosen as six segments of comparable length (450 ± 50 m) within the CBD with a high density of retail and food outlets. Suburban segments covered a broad range of SES by census data, with sections of 450 ± 50 m centered on areas of similarly high outlet density.

The area deprivation measure used was calculated for small areas from 2013 census data [22]. The suburban areas selected ranged from 1 (least deprived decile) to 9 (second-most deprived decile). Google Maps was used to select 450 ± 50 m stretches of each street that had a high density of retail and food outlets. Boundaries were marked by street numbers or intersections.

### Data collection

A data collection tool was developed to standardize the data collection process (see http://www.otago.ac.nz/wellington/otago114171.pdf). It provided categories into which visual data could be recorded and aimed to include all alcohol-related items that a typical pedestrian could be exposed to, including venues, advertising, health promotion and regulation items, and visible consumption of alcohol. It was refined during pilot trials using both on-street and GSV survey methods. Criteria for classifying items were developed to improve inter-rater reliability (see http://www.otago.ac.nz/wellington/otago114170.pdf). The final definitions used for defining venues, marketing and other alcohol-related materials are shown in Table 1. Maps of street segments, the data collection tool and criteria were provided to the observer teams.

**Table 1:** Definitions used for classifying alcohol-related venues, marketing and other materials

Term	Details
Alcohol retail venues	Retail venues with visible evidence of alcohol sales.
Other retail venues	Retail venues with no evidence of alcohol sales.
Venue-associated brand marketing	Advertisement or marketing of alcohol or alcohol beverage brands related to a venue that sold alcohol e.g., a poster for a brand of beer on the outside wall of a bar.
Isolated brand marketing	As above but not related to a venue that sold alcohol.
Outlet marketing	Advertisements or marketing promoting drinking or exhibiting consumption of alcohol. General and not consisting of a brand or corporate aspect e.g., a “Happy Hour” sign or “BYO” (bring-your-own) sign.
Alcohol health-promotion materials	Materials promoting relatively safe consumption of alcohol e.g., host responsibility statements stating approaches to dealing with, intoxicated and underage people.
Alcohol regulatory materials	Materials pertaining to the regulation of alcohol consumption e.g., legislated signage stating identification requirement for all alcoholic purchases for those under 25 years.
Visible drinkers	Instances of people drinking what is likely to be alcohol beverages (on a balance of probabilities).
Alcohol-related litter	Trash or litter that is thought to be alcohol-related (on a balance of probabilities), e.g., an empty beer can or bottle.
Other pro-alcohol materials	Any other material promoting alcohol consumption that does not fall into any of the above categories.

Each street segment was surveyed twice using on-street surveying, and twice using GSV, within a two week period in April 2015 (mid-autumn in New Zealand). Surveys were completed by pairs of researchers, who reached agreement before any data item was recorded. No pair surveyed the same street segment twice, regardless of the observation method, and researchers were blinded to other pairs’ results. Observers were almost exclusively New Zealand raised and 20–30 years of age. Ethics approval for this project was obtained in April 2015, via the Department of Public Health Ethics Review process as per the standard University of Otago process for Category B approval. No participants were involved.

### On-street surveys

On-street surveys were undertaken in daylight hours on weekdays, in fine weather, with all surveys completed between 1430 h and 1730 h. Researchers began at the boundary of their allocated street segment, walked down one side of the street and returned along the opposite side, while observing the streetscape and recording relevant items using the data collection tool. The observers regularly looked back along the street, observed structures on traffic islands and noted anything on the opposite side of the street that could only be seen from afar (e.g., large signs high on buildings). The time taken to complete each survey was noted, and Google Maps was later used to calculate driving time to survey locations from a base point (excluding traffic effects).

### Google Street View surveys

These surveys were completed by observing GSV images of the selected street segments, across the same two-week period of April 2015. Researchers began each survey at the boundary of their allocated street segment, navigated down the street in one direction before returning back down the street to their starting point. Data were gathered using images taken from the road lane closest to the footpath where possible, and if images from a footpath view were available these were also used. The angle of view was altered regularly to view structures in the middle of the street and ensure the entire height of buildings on the opposite side of the street could be seen, as well as to regularly look back and forward down each street to see any signs not visible from a straight-on viewing angle. The zoom tool was used to provide a closer view of signage where appropriate, to match what a pedestrian could easily read. The range of image dates and most frequent dates of image capture were recorded for each street segment, along with time taken to complete each survey.

### Statistical analyses

Statistical analyses were conducted using R (v 3.2, R Institute, Vienna, Austria). The reliability of observations both between pairs (inter-observer-team reliability) and between methods (on-street vs GSV) was measured by calculating the average intra-class correlation coefficient (ICC) score using two main variables: detection of venues with evidence of alcohol sale and total number of alcohol-related advertisements. ICC results were interpreted with reference to common kappa coefficient cut-offs, as discussed in the literature and as follows: 0.00–0.20 slight agreement; 0.21–0.40 fair; 0.41–0.60 moderate; 0.61–0.80 substantial and 0.80–1.00 almost perfect [23].

Times taken to complete the surveys were compared between the on-street and GSV methods. To conduct these analyses, the average time taken for each location/method (as two teams completed each survey for a particular location/method), and these times were compared between on-street and GSV methods using a paired *t*-test. Two tests were conducted: one comparing GSV survey time with on-street survey time (no travel component); and a second one which was the same but with including the travel component.

## Results

In 23.3 h of street surveying by both methods across all 12 street segments, we identified 403 retailers with evidence of alcohol sales and 1161 items of alcohol-related communication. Of the latter, 1028 (89 %) were for alcohol marketing and 133 (11 %) were for alcohol-related health promotion and alcohol regulation (Table 2). The collective knowledge by observer pairs of alcoholic brand names, common signage and advertising practices was generally sufficient to categorize all items encountered during surveying. While most of the relevant GSV images were from February 2015, in three CBD and three suburban street segments some of the images were from 2008 or 2009.

**Table 2:** Comparison of on-street observation and the use of GSV for alcohol-related imagery in 12 street segments (Wellington, New Zealand, April 2015)

	On-street			GSV			Difference between methods		
Characteristic	Total identified [A]	Mean [B]	SD	Total identified [C]	Mean [D]	SD	Total identified [A]-[C]	Mean [B]-[D]	% difference [C]/[A]
Alcohol retail venues	268	11.1	10.8	135	5.6	5.5	133	5.5	50 %
Other retail venues	400	16.7	10.1	440	18.3	11.1	−40	−1.6	110 %
Isolated brand marketing	99	4.1	9.0	15	0.6	1.2	84	3.5	15 %
Venue associated brand marketing	296	12.3	12.1	153	6.4	6.3	143	5.9	52 %
Outlet marketing	633	26.4	25.7	214	8.9	8.9	419	17.5	34 %
Alcohol health promotion materials	20	0.8	1.5	1	0.0	0.2	19	0.8	5 %
Alcohol regulatory	113	4.7	5.3	32	1.3	1.8	81	3.4	28 %
Visible drinkers	96	4.0	9.5	12	0.5	1.9	84	3.5	13 %
Alcohol-related litter	24	1.0	2.0	1	0.0	0.2	23	1.0	4 %
Other pro-alcohol	6	0.3	0.6	1	0.0	0.2	5	0.2	17 %

Note. Total values are the sum of all items identified across all 12 street segments surveyed. Mean values are the number of items observed per 400 ± 50 m street segment. *SD* standard deviation. Refer to the main text and Table 1 for detail on characteristics

### Venues with evidence of alcohol sales

**Fig. 1:**
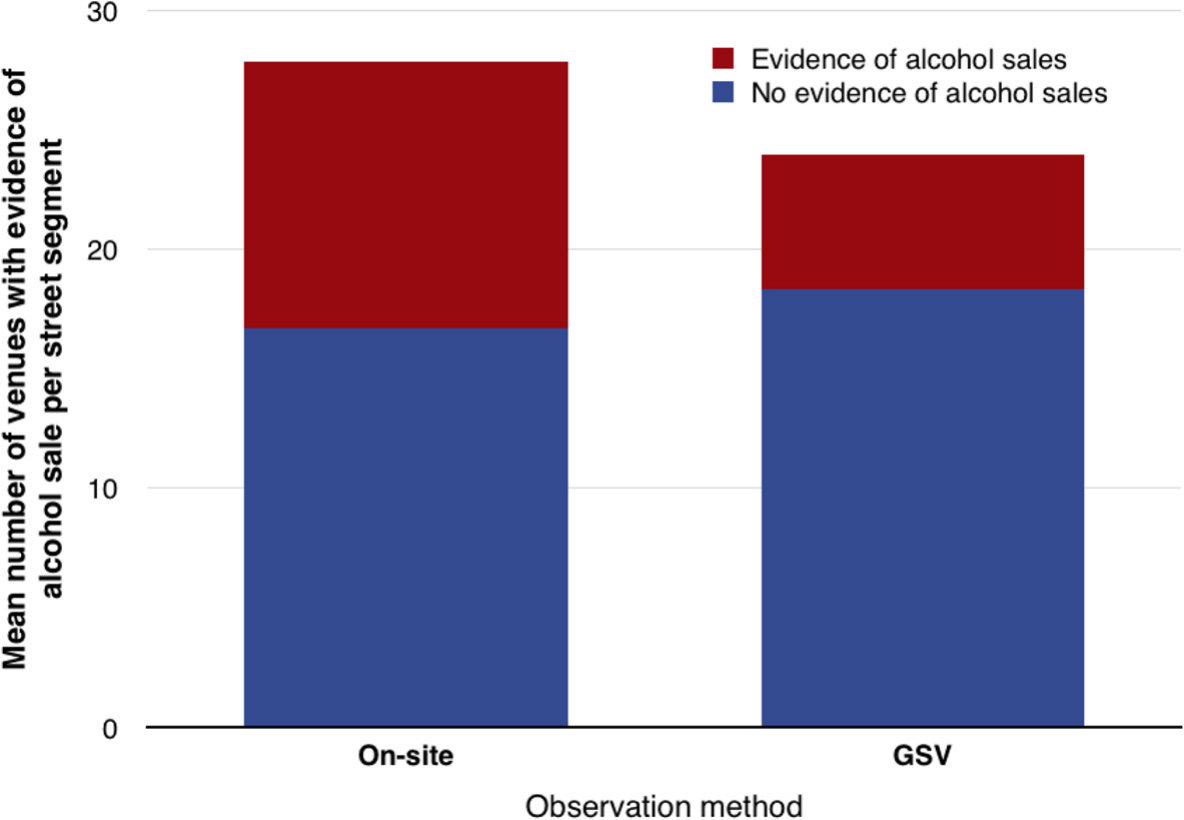
Mean number of venues per street segment with evidence of alcohol sale by observation type (on-street observation vs use of GSV)

On-street observers found a mean of 11.1 alcohol retail venues and 16.7 other venues per street segment (total mean: 27.8 venues; total venues: 668). Corresponding data for GSV showed a mean of 5.6 alcohol retail venues and 18.3 other venues detected per street segment (total mean: 24 venues; total venues: 575). The relative proportion of alcohol retail venues to other venues found varied across the methods: 40 % of all venues by on-street observation and 23 % by GSV (Fig. 1).

### Alcohol-related advertising

**Fig. 2:**
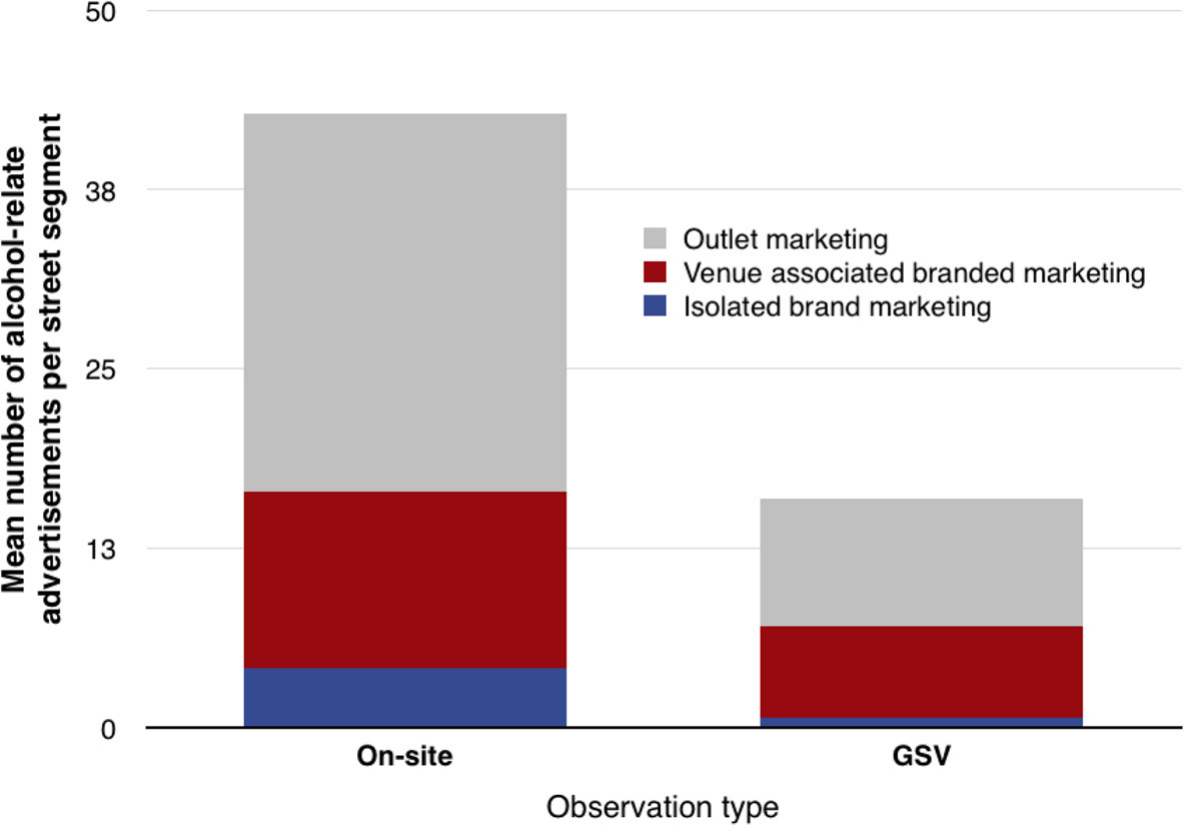
Alcohol-related advertisements, outlet marketing, and alcohol brand marketing per street segment by method of observation (on-street observation vs use of GSV)

Of all alcohol-related advertising, outlet marketing was the most frequently observed, followed by venue-associated brand marketing. Isolated brand marketing was seen less frequently (Fig. 2). This was almost universally the pattern across locations and across both observation types. Overall, GSV was less effective at detecting all types of alcohol-related advertising than on-street observation, picking up 37 % of the amount detected by on-street observers.

### Health promotion activity and alcohol regulation

**Fig. 3:**
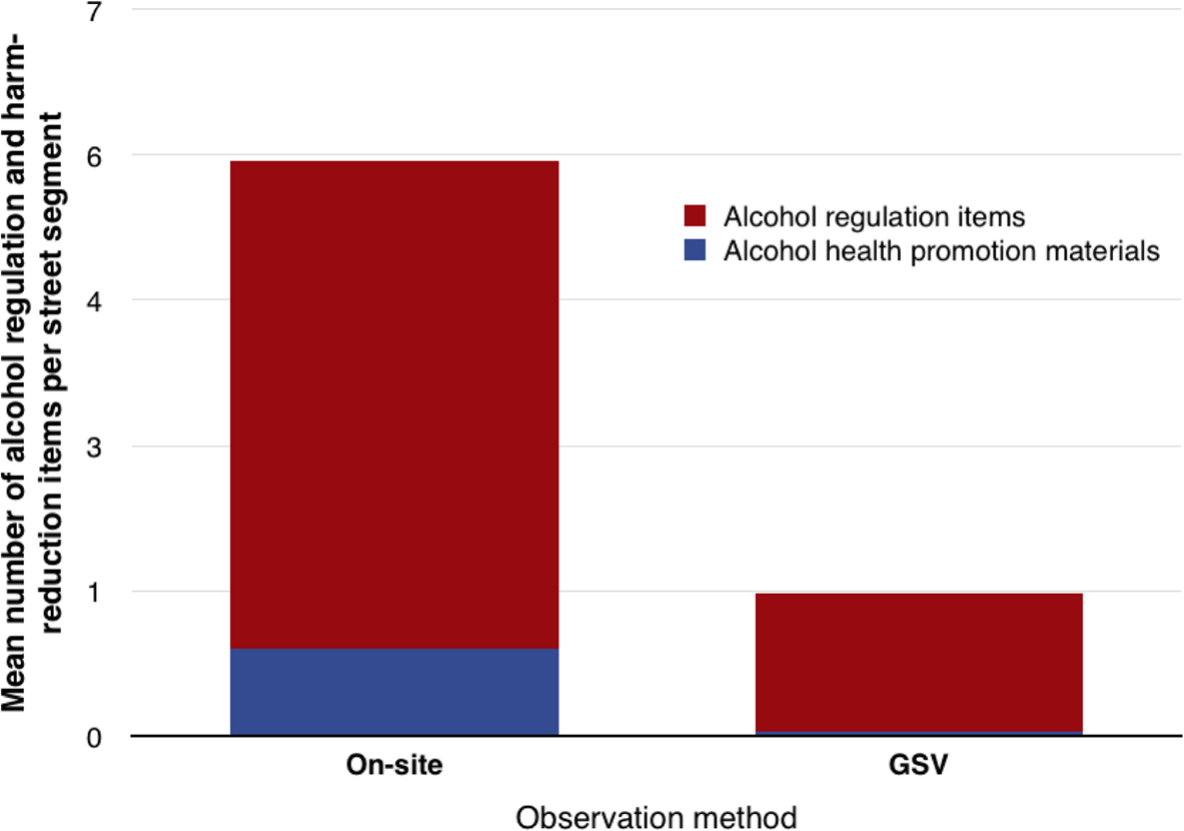
Mean number of alcohol regulation and health promotion items per street segment by method of observation (on-street observation vs use of GSV)

There was a marked lack of visible alcohol-related health promotion materials in all street segments surveyed, with a mean of 0.8 items seen per street segment by on-street observation. GSV was very poor for detection of these items (Fig. 3). Similarly, GSV was able to identify only 28 % of the alcohol regulation items seen by on-street observers, revealing a mean 1.3 items per street segment compared to 4.7 items per segment found by on-street data collection.

### Visible alcohol consumption

On-street observation revealed a mean of 4.2 visible drinkers, though considerable variation was found across locations, with all the drinkers seen in just two street segments: the ‘hospitality areas’ of Cuba St and Courtney Place. GSV detected only 13 % of visible drinkers when compared to on-street data collection (but GSV is likely to have been across a wider daytime time period than on-street observation).

### Survey time comparisons

The time taken to complete on-street observation and GSV observation was analyzed across all street segments, with travel times calculated using Google Maps and excluding traffic effects.

On-street observation was performed in a mean of 32 min per street segment (range: 11–75 min, median: 26 min), which increased to a mean of 39 min (range: 17–83 min, median: 34 min) when including travel time (travel time range: 1–15 min, median: 6 min). GSV surveying was completed in a mean of 29 min per street segment (range: 9–47 min, median: 32 min). The survey times per street segment were not significantly different between GSV and on-street survey methods when travel time was not included (mean GSV time 3.5 min faster, 95 % confidence interval (CI): −5.9, 12.9; *p* = 0.429); factoring in travel time showed a modest advantage for GSV over on-street observation (mean GSV time 10.2 min faster, 95 % CI: 0.3, 20.0; *p* = 0.045).

### Inter-observer and inter-method reliability testing

A high degree of reliability was found between pairs of on-street observers in the detection of alcohol retail venues. The average measure ICC was 0.93 (95 % CI: 0.78 to 0.98). Similarly, a high degree of reliability was found between pairs of GSV observers in detection of the alcohol retail venues, with an ICC calculated as 0.85 (95 % CI: 0.49 to 0.96).

Similarly, a high degree of reliability was found between pairs of on-street observers in the detection of alcohol-related advertising, with an ICC calculated as 0.92 (95 % CI: 0.72 to 0.98). In contrast, only a moderate degree of reliability was seen between pairs of GSV observers in detection of the same class of items. The average measure ICC was 0.60 (95 % CI: −0.35 to 0.88).

Inter-method variability revealed a moderate degree of reliability between on-street and GSV observation in detection of alcohol retail venues (ICC = 0.50, 95 % CI: −0.05 to 0.82). But the same analysis performed for alcohol-related advertising suggested relatively poor reliability between on-street and GSV observation, with an ICC of 0.04 (95 % CI: −0.53 to 0.60).

## Discussion

### Main findings and interpretation

In this study, GSV was found to be generally less sensitive than on-street observation in identifying various aspects of alcohol-related content in the urban streetscape environment. The sensitivity difference between GSV and on-street surveying was largest when looking for visible drinkers, followed by alcohol-related advertising, and then venues with evidence of alcohol sales. The lower sensitivity of GSV for visible drinkers may be due to the small size of alcoholic beverages in the imagery. Similarly, some advertising materials are difficult to identify in GSV images with limited resolution and ability to zoom. This picture was supported by the subjective experience of researchers: small item detection was felt to be difficult using GSV, whereas large static objects were generally easy to identify and categorize (although GSV observation still found only 86 % of the venues that on-street observation found). Compared to visible drinkers and alcohol-related advertising, there was greater concordance between the total number of venues identified between both methods, suggesting that GSV may provide useful data on large, static components of the built environment (e.g., alcohol retail venues and large signs).

While surveying with GSV showed a decreased survey time compared to the on-street method, it was only statistically significant when travel time was also included in the analysis. However, it is plausible that further familiarization with the technique could enable greater reduction in time taken to survey compared to surveying on foot. It should also be noted that in this study the researchers’ workplace was based within six km of the street segments, so travel times were relatively small.

Another reason for the difference in results is that GSV’s in-built blurring algorithm, although designed to maintain anonymity of people photographed, often blurs items close to the face which then limits the detection of people consuming alcoholic beverages. GSV also performs automatic blurring of a large proportion of signs, which is a feature intended to blur vehicle license plates (at least in the New Zealand setting). This seems to have rendered detection and categorization of some signage difficult.

The range in GSV image capture dates and times may account for some of the observed variation when compared with on-street observation. Though the majority of imaging was taken within three months of the observations, the oldest images were from 2008. It is also possible that some venues, signage or seating areas for drinkers may have changed even in the few months between the dates of image capture used in our GSV surveys and on-street surveys. In terms of the wider use of GSV, if it was to be used for monitoring of policy, e.g., advertising regulations, then frequent new imaging would be required to ensure up to date information is provided. Within the region studied, images had generally been updated on two occasions since the initial establishment of GSV coverage in 2008.

The time of day of GSV image capture was also variable. Even within daylight hours the presence of portable signage and seating can vary, and without knowing exact times of image capture it is impossible to truly match on-street observation times to image capture times when comparing survey methods. This may particularly affect the number of visible drinkers seen by GSV, compared to that seen by the 1430 h to 1730 h on-street observation, as there is likely to be a higher prevalence of drinking in this mid-late afternoon period, compared to across the whole day.

Images captured on GSV may be further limited by the means by which GSV images are acquired. Vehicles used to capture GSV images may be unable to access pedestrian-only areas of streets or areas with difficult road access, resulting in suboptimal image angle or quality.

The fact that GSV images were also taken during daylight hours makes it broadly comparable to on-street observations, but exclusive use of daylight data may limit our ability to draw universally applicable conclusions. Since many bars and restaurants neither open nor display portable signage until late afternoon, and not all our data collection overlapped with this period, we suspect that our findings may not reflect the maximal amount of exposure, and hence the maximum potential to influence behaviors. However, exposure to large, static alcohol and tobacco-related imagery in the streetscape is unlikely to be significantly affected by this.

### Study strengths and limitations

A notable strength of this study was it being the first (to our knowledge) to rigorously compare GSV with on-street observation for alcohol-related imagery in the urban streetscape. It was able to do this for a wide range of imagery and in a range of different types of streets and suburbs. The observers were also all very familiar with alcohol-related imagery in the urban environment studied. The study results should have reasonable generalizability to other developed country urban environments – but some cities might have different alcohol-related imagery (e.g., if they have legal constraints on alcohol advertising and sponsorship – which hardly exist in the New Zealand setting).

Nevertheless, this study could have been more rigorous if the time of day, day of week and season were exactly matched between the GSV image date and the on-site observations (this was not feasible in this study for logistic reasons). Improvements could also have been made in the standardization of street segment lengths for surveying (for example, Google Maps universally rounds distances to the nearest 50 m, meaning we were only able to standardize street length to 400 ± 50 m). Variations in the lengths of street segments selected for surveys may have contributed to some of the variation in counts of observed environmental factors across street segments.

### Potential future research

Future study in this area could also take into account both the need for prior knowledge of signage and brands in order to identify alcohol-related advertising, and the time of data collection. The level of observer knowledge could be a consideration for future research attempting to use similar methods. Prior knowledge was especially helpful when using GSV, as signs were often blurry, unclear or of poor resolution. The requirement of background knowledge may suggest that for surveys using GSV in international or cross-cultural contexts, additional observer training might be important. Furthermore, our study was limited to data collection during daylight hours only, and further studies could include evening periods.

It is worth noting that “footpath views” in GSV were occasionally available in our study, were of a much higher image quality and yielded a more accurate representation of the pedestrian experience. If this footpath view were to become more universal in urban settings, and image quality and frequency were increased, GSV could become be a more valid tool for surveying urban streetscapes for health determinants such as alcohol-related imagery.

Further studies could also investigate to see if the exclusion of older GSV images was a way to improve the reliability of evidence, compared to on-street observation.

## Conclusions

At this stage, it seems that the use of GSV is unlikely to be suitable for the comprehensive study of the influences on alcohol consumption in the urban streetscape. Its limitations in terms of low sensitivity and inter-observer reliability are such that it is a fairly poor substitute for on-street observation. However, GSV may still have significant merit for the surveying of large, static alcohol-related components of the built environment (e.g., venues and large advertising signs), and may be more time efficient than traditional on-street observation measures, especially when used to collect data across a wide geographical area. Furthermore, GSV might be a more useful research tool in settings with better image quality (such as more ‘footpath views’) and more regularly updated GSV imagery.

## Abbreviations

CBD, central business district; GSV, Google Street View; ICC, intra-class correlation coefficient; SES, socioeconomic status
